# Life after bone infection: a retrospective comparison of quality of life in patients with periprosthetic joint infection and fracture-related infections

**DOI:** 10.1186/s13018-025-06427-2

**Published:** 2025-11-19

**Authors:** J. Frese, L. Schwake, A.-P. Schulz, S. Schaeffer, U.-J. Gerlach, C. Grimme

**Affiliations:** 1 Department of Septic Bone and Joint Surgery, BG Klinikum Hamburg, Bergedorfer Strasse 10, 21033 Hamburg, Germany; 2 Section of Medicine, Universität zu Lübeck, Ratzeburger Allee 160, 23562 Lübeck, Germany; 3 Center for Clinical Research, BG Klinikum Hamburg, Bergedorfer Strasse 10, 21033 Hamburg, Germany

**Keywords:** Periprosthetic joint infection, Fracture-related infection, Health related quality of life, EQ-5D-3L, Retrospective study, Patient-reported outcomes

## Abstract

**Background:**

Periprosthetic joint infections (PJI) and fracture-related infections (FRI) are among the most severe complications in orthopaedic trauma surgery. In addition to a significant medical burden, both conditions are associated with prolonged hospitalisation, increased morbidity, increased costs and reduced health-related quality of life (HRQoL). While the clinical course of implant-associated infections is well characterised, comparative data on patient-reported outcomes remain limited.

**Objective:**

This study aimed to compare HRQoL in patients treated for PJI versus FRI, and to analyse associations between clinical parameters such as ASA classification, length of stay (LOS), and number of revision procedures.

**Methods:**

In this retrospective, monocentric cohort of 61 patients with microbiologically confirmed implant associated infections treated in 2021 completed the EQ-5D-3 L and EQ-VAS questionnaires by post in 2023 (response rate 37.9%). The cohort comprised 30 PJI and 31 FRI patients. The primary outcome was EQ-VAS. Secondary outcomes included the mean EQ-5D-3 L domain level (range 1–3; higher values indicate worse health). Clinical data were extracted from hospital records and analysed using parametric or non-parametric tests.

**Results:**

Patients with PJI reported significantly reduced self-perceived health status (EQ-VAS: 50.4 ± 18.9) than those with FRI (61.9 ± 19.3; *p* = 0.036). The mean EQ-5D-3 L domain level was 1.83 ± 0.37 in PJI versus 1.65 ± 0.39 in FRI (*p* = 0.061). PJI patients had significantly longer hospital stays (82.9 vs. 33.1 days; *p* < 0.001) and higher ASA scores (2.71 vs. 2.35; *p* = 0.01).

**Conclusion:**

PJI was associated with significantly poorer HRQoL than FRI, possibly reflecting greater comorbidity and more invasive treatment. These findings emphasise the importance of interdisciplinary, patient-centred care, as well as the need for prospective studies to clarify HRQoL trajectories and mediators following implant-associated infections.

**Supplementary Information:**

The online version contains supplementary material available at 10.1186/s13018-025-06427-2.

## Introduction

Implant-associated infections, including periprosthetic joint infections (PJI) and fracture-related infections (FRI), are among the most severe complications in orthopaedic and trauma surgery. Despite their relatively low incidence rates of 23.8 and 10.7 per 100,000, respectively, in Germany in 2019, these infections exert a substantial clinical and systemic impact [1–3]. They often lead to prolonged hospitalisations, repeated revision surgeries, long-term antibiotic therapy, and considerable healthcare costs [4–6]. The complexity of their treatment is further amplified by the need for individualised surgical approaches, especially in the presence of biofilm-forming pathogens or soft tissue compromise [7].

Recent reviews have also emphasised that infection-associated bone loss often requires advanced reconstructive procedures to restore stability and function [8], while current management concepts of adult osteomyelitis emphasise the importance of staged debridement, microbiological control and reconstruction principles [9].

Beyond the surgical and medical challenges, patients with implant-associated infections frequently experience long-term impairments in mobility, autonomy, and psychosocial functioning [10–12]. Anxiety and depressive disorders are more prevalent in this population and are known to negatively influence recovery trajectories and reinfection risk [2, 11]. A prospective study by Knebel et al. reported high psychosocial distress in patients with knee PJI, underlining the multifactorial impact of the condition [13]. Similarly, Krenn et al. found that many patients perceive PJI as a chronic, identity-disrupting illness with profound effects on social roles and quality of life [14]. These findings align with large-scale data from Cooper et al., which demonstrated that patients experience persistent impairments in mobility, pain, and emotional well-being even after successful PJI treatment [15].

Although fracture-related infections (FRI) generally entail a lower systemic burden, recent studies show that complex, fracture-related infections can substantially reduce health-related quality of life (HRQoL), especially in cases involving multiple revisions or delayed healing [5, 16]. He et al. further emphasised that the long-term consequences of FRI extend beyond physical recovery to include emotional and social dimensions [17].

Despite this growing recognition, patient-reported outcomes (PROs) remain underrepresented in clinical outcome research on implant-associated infections. While several studies have evaluated HRQoL following PJI or FRI separately, direct comparisons between the two infection types are rare. Furthermore, the relationship between clinical variables, such as the American Society of Anesthesiologists (ASA) classification, length of hospital stay, and number of revision surgeries, and HRQoL outcomes has not yet been systematically investigated.

The EQ-5D-3 L instrument is a validated, widely used tool for measuring HRQoL in orthopaedic, oncologic, and chronic care populations [18–20]. However, comparative analyses between PJI and FRI using the EQ-5D remain limited in the literature.

### Objective

This study aimed to evaluate and compare the health-related quality of life (HRQoL) in patients with periprosthetic joint infection (PJI) and fracture-related infection (FRI) using the EQ-5D-3 L instrument. A secondary objective was to explore potential differences in clinical parameters, such as ASA classification, length of hospital stay, and revision surgery rates, between the two groups and to examine whether these factors may be associated with variations in HRQoL.

## Methods

This retrospective monocentric cohort study was conducted at the Department of Septic Orthopaedic and Trauma Surgery at BG Klinikum Hamburg, a Level I trauma and tertiary referral centre in Germany. The study was approved by the ethics committee at the University of Regensburg (reference number 23-3468-101). All participants provided written informed consent by completing and returning a postal questionnaire. The study aimed to evaluate the health-related quality of life (HRQoL) of patients with periprosthetic joint infections (PJI) or fracture-related infections (FRI) who were treated at the institution in 2021.

**Fig. 1:**
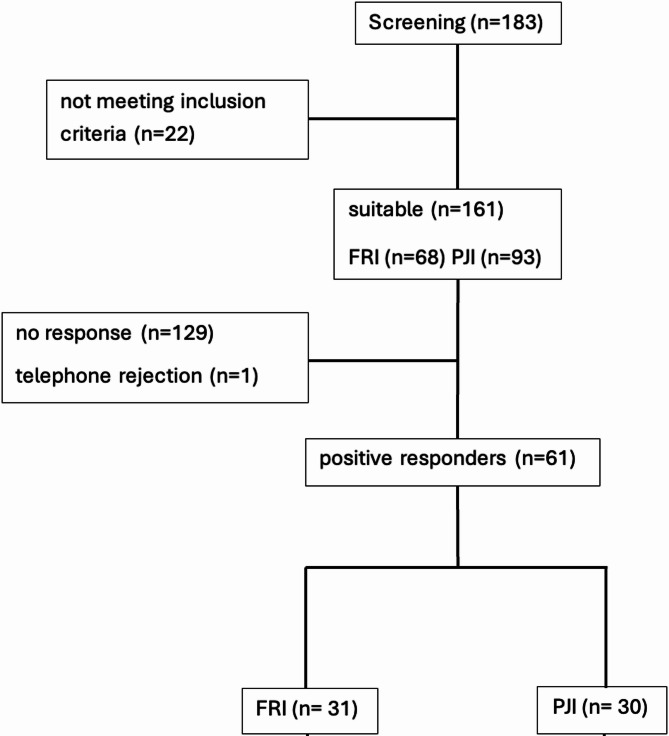
Flowchart of patient recruitment. Of 183 screened patients, 161 were eligible (93 PJI, 68 FRI). After 129 non-responses and 1 refusal, 61 patients were included (30 PJI, 31 FRI)

Patients were identified through retrospective screening of electronic hospital records. Eligible patients were at least 18 years old and had a microbiologically confirmed diagnosis of either PJI or FRI. Classification followed the Musculoskeletal Infection Society (MSIS) and the International Consensus Meeting Criteria (ICM) and was complemented by clinical principles from the Pro-Implant Foundation. These principles consider infection timing, implant stability, and pathogen identification in therapeutic planning [7, 13]. Exclusion criteria included incomplete documentation, unreachability, or lack of consent. Of the 183 patients who were screened, 161 met the inclusion criteria (PJI: *n* = 93; FRI: *n* = 68). In 2023, these patients were invited to participate via mail. A total of 61 patients (PJI: 30; FRI: 31) returned the questionnaire, yielding a response rate of 37.9%. The German validated EQ-5D-3 L questionnaire, including the EQ-VAS, is reproduced in Supplementary Material 1. The analyses followed a complete-case approach, no imputation was performed. Variable-specific denominators (n) reflect missingness at the item-level. Figure 1 illustrates the recruitment process.

Data collection proceeded in two stages. First, clinical and demographic data, including age, sex, anatomical site, microbiological findings, ASA (American Society of Anesthesiologists Physical Status Classification System) classification, length of hospital stay (LOS), number of revision surgeries, recurrent infection after initial quiescence, amputation, and in-hospital mortality, were extracted from electronic records. Second, educational background and health-related quality of life (HRQoL) were assessed using a postal EQ-5D-3 L questionnaire, which included the EQ visual analogue scale (EQ-VAS). Returned questionnaires were manually checked for completeness and plausibility. Only fully completed responses were included in the final analysis (complete-case analysis), and no imputation was performed. For each patient two distinct time intervals were calculated: The time from index surgery to infection onset (defined as the period between primary implantation or fracture fixation and microbiologically confirmed infection) and the time from the last index or revision surgery to survey completion (reflecting the time elapsed between the most recent surgical intervention and the HRQoL assessment). Both intervals are reported as median (IQR), mean ± SD and range (minimum – maximum).

The EQ-5D-3 L instrument measures five dimensions (mobility, self-care, usual activities, pain, and anxiety) on a three-level scale (1 = no problems, 2 = some problems, 3 = extreme problems). For the present analysis, the primary outcome was EQ-VAS. Secondary outcomes included the mean EQ-5D-3 L domain level, calculated as the arithmetic mean across the five domains (range 1–3, higher values indicating worse health). Utilities were not computed. The wording of the validated German EQ-5D-3 L is provided in Supplementary Material 1.

The EQ-5D-3 L has been validated for use in orthopaedic and infection-related settings, correlating well with clinical outcomes and patient-reported pain and function [16, 18].

Additionally, the EQ-VAS records patients’ subjective health status on a scale from 0 (worst imaginable health) to 100 (best imaginable health). The EQ-5D is considered suitable for postal self-reporting and has been successfully applied in chronic PJI cohorts where it shows a strong correlation with pain, function and mental health dimensions [15, 19].

Statistical analyses were conducted using R (version 2024.12.1 + 563). Continuous variables were tested for normality using the Shapiro-Wilk test and equality of variances with Levene’s test. Depending on assumptions, groups were compared using two-sample t-tests (equal variances) or Wilcoxon rank-sum test (non-parametric). Categorical variables were analysed using Pearson’s chi-square test with Yates’ continuity correction or Fisher’s exact test as appropriate.

Effect sizes were reported as mean differences with 95% confidence intervals for continuous outcomes and as Cramér’s V (bias-corrected) for categorical variables. For skewed continuous distributions, Wilcoxon r was also calculated.

To address baseline imbalances and potential confounding, we pre-specified a multivariable linear regression with EQ-VAS as the dependent variable and infection type (PJI vs. FRI) as the main exposure, adjusting for age, sex, ASA score and time from last index/revision surgery to survey. Length of stay and number of revisions were considered potential mediators and were therefore excluded from the primary model. An exploratory model including these variables is presented in the supplement. Results are reported as adjusted b coefficients with 95% confidence intervals.

Due to the retrospective and exploratory nature of the study, no formal sample size or power calculation was performed. However, including all available patients resulted in two balanced groups (PJI: *n* = 30; FRI: *n* = 31), which allowed for meaningful comparisons. Larger sample sizes would be required for reliable subgroup analyses or multivariate modeling.

## Results

**Table 1:** Comparison of clinical, surgical, and microbiological characteristics between FRI and PJI groups

	FRI (*n* = 31)	PJI (*n* = 30)	*p* - value
Sex (*n* = 61)			
Female	8 (25.8)	12 (40.0)	0.364
Male	23 (74.2)	18 (60.0)	
Age (years) (*n* = 61)			
Mean ± SD	61.13 ± 14.22	67.97 ± 9.79	0.033*
Min – Max	29–86	49–86	
Side (*n* = 60)			
Left	12 (40.0)	17 (56.7)	0.301
Right	18 (60.0)	13 (43.3)	
ASA 1st Revision (*n* = 59)			
Mean ± SD	2.35 ± 0.55	2.71 ± 0.53	0.018*
1	1 (3.2)	0	
2	18 (58.1)	9 (32.1)	
3	12 (38.7)	18 (64.3)	
4	0	1 (3.6)	
One Reinfection (*n* = 60)	15 (48.3)	19 (65.5)	0.281
Two Reinfections (*n* = 59)	14 (45.2)	18 (64.3)	0.226
Revision surgeries (*n* = 60)	19 (61.3)	17 (58.6)	1.000
Revision strategy (*n* = 57)			
One-stage	9 (29.1)	5 (19.2)	0.584
Two-stage	22 (70.9)	21 (80.7)	
Microbiological Results (*n* = 61)			
*S. epidermidis*	12 (38.7)	18 (60.0)	0.160
*S. aureus*	15 (48.4)	9 (30.0)	0.227
*E. faecalis*	2 (6.4)	5 (16.6)	1.000
*S. capitis*	4 (12.9)	2 (6.6)	0.671
Time: Index Surgery to Infection (months) (*n* = 61)			
Mean ± SD	12.68 ± 24.00	32.16 ± 59.04	0.144
Min - Max	1–120	0.5–216	
Length of hospital stay (days) (*n* = 61)			
Mean ± SD	33.13 ± 26.12	82.90 ± 81.17	0.0003*
Min - Max	8–126	5–341	
EQ-5D-3 L Dimensions			
Mobility (*n* = 53)	1.78 ± 0.51	1.81 ± 0.50	0.830
Self-care (*n* = 53)	1.33 ± 0.68	1.50 ± 0.51	0.101
Usual activities (*n* = 52)	1.78 ± 0.58	1.92 ± 0.57	0.377
Pain (*n* = 51)	1.93 ± 0.39	2.08 ± 0.57	0.258
Anxiety (*n* = 53)	1.48 ± 0.51	1.77 ± 0.71	0.150
Patient level mean EQ-5D-3 L domain level (*n* = 48)			
Mean ± SD	1.65 ± 0.39	1.83 ± 0.37	0.061
Min - Max	1–2.6	1–2.4	
General health status (EQ-VAS) (*n* = 52)			
Mean ± SD	61.89 ± 19.31	50.44 ± 18.89	0.036*
Min - Max	20–95	10–80	
Amputation (*n* = 60)	1 (3.2)	2 (7.4)	0.606
Mortality until survey completion (*n* = 61)	4 (12.9)	2 (6.6)	0.672
Smoking status (*n* = 52)			
Current	7 (26.9)	3 (11.5)	0.291
Former	10 (38.5)	12 (46.1)	0.779
Never	9 (34.6)	11 (42.3)	0.776
General illness			
Own illness (*n* = 44)	15 (60.0)	15 (78.9)	0.313
Family illness (*n* = 39)	16 (76.2)	15 (83.3)	0.702
Other illness (*n* = 25)	5 (35.7)	5 (45.5)	0.697
Higher education (*n* = 51)	26 (96.3)	20 (83.3)	0.175
Academic degree (*n* = 51)	9 (34.6)	6 (24.0)	0.600

This table presents a comparative analysis of patients with fracture-related infections (FRI) and periprosthetic joint infections (PJI), including demographic data, ASA classification, reinfection rates, revision strategy, and microbiological findings. Statistically significant values (*p* < 0.05) are marked with an asterisk (*). FRI = Fracture-related Infection. PJI = Periprosthetic Joint Infection. Values are mean ± SD or n (%), as indicated

A total of 61 patients (30 PJI, 31 FRI) completed the EQ-5D-3 L questionnaire in 2023, corresponding to a response rate of 37.9%. All patients had been treated in 2021. Responders (*n* = 61) and non-responders (*n* = 100) did not differ significantly in age, sex ASA classification or infection type (all *p* > 0.05; data not shown).

The mean age was higher in the PJI group (68.0 ± 9.8 years) than in the FRI group (61.1 ± 14.2 years; *p* = 0.033). The gender distribution was comparable (Table 1).

Regarding time intervals, the mean time from index surgery to infection onset was 12.7 ± 24.0 months in the FRI group (median 4, IQR 2–12, range 1-120) and 32.2 ± 59.0 months in the PJI group (median 9, IQR 3–24, range 0.5–216), with no statistically significant difference (*p* = 0.144).

The mean time from index surgery to survey completion was 115.6 ± 92.4 months (median 49, IQR 42–163, range 33–372; *n* = 30) in the PJI group and 44.3 ± 20.6 months (median 37, IQR 29–58; range 18–84; *n* = 31) in the FRI group. When calculated from the last revision surgery the mean time to survey completion was 27.8 ± 11.5 months (median 32, IQR 26–36, range 3–39) in PJI and 30.3 ± 10.2 months (median 30, IQR 23–37, range 4–56) in FRI.

Most infections affected the lower extremities. In the FRI group (*n* = 31), infections frequently involved the tibia (12/31; 38.7%) and femur (5/31; 16.1%). Additional cases affected the knee (4/31; 12.9%), ankle (2/31; 6.5%), hip (2/31; 6.5%) and tarsal bones (2/31; 6.5%), shoulder (1/31; 3.2%), radius (1/31; 3.2%), humerus (1/31; 3.2%) and thoracic vertebra (1/31; 3.2%). Overall, 87.1% (27/31) of FRI cases affected the lower limb.

In the PJI group (*n* = 30), most commonly affected joints were the knee (13/30; 43.3%) and the hip (10/30; 30.0%), followed by the shoulder (3/30; 10.0%), ankle (3/30; 10.0%), and hand (1/30; 3.3%).

**Fig. 2:**
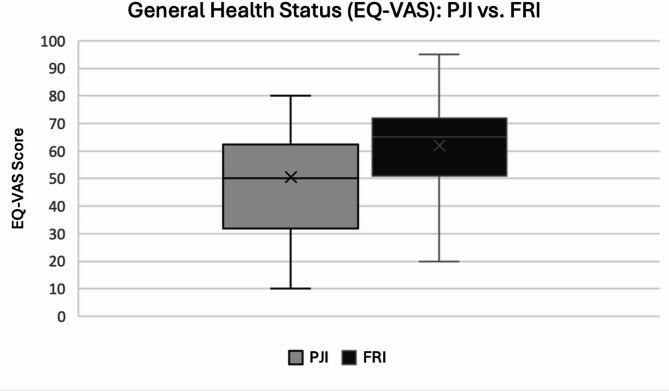
EQ-VAS by infection type (0-100; higher = better). Boxes show the interquartile range; horizontal line = median; “x” = group mean; whiskers = min-max. Group sizes: PJI *n* = 25, FRI *n* = 27. Means *±* SD: PJI 50.4 *±* 18.9, FRI 61.9 *±* 19.3; *p* = 0.036. Analyses followed a complete-case approach; variable-specific denominators reflect item level missingness

As shown in Fig. 2, the EQ-VAS scores were significantly higher in the FRI group (61.89 ± 19.31) than in the PJI group (50.44 ± 18.89; *p* = 0.036), corresponding to an unadjusted mean difference of 11.45 (95% CI 0.81–22.09).

**Fig. 3:**
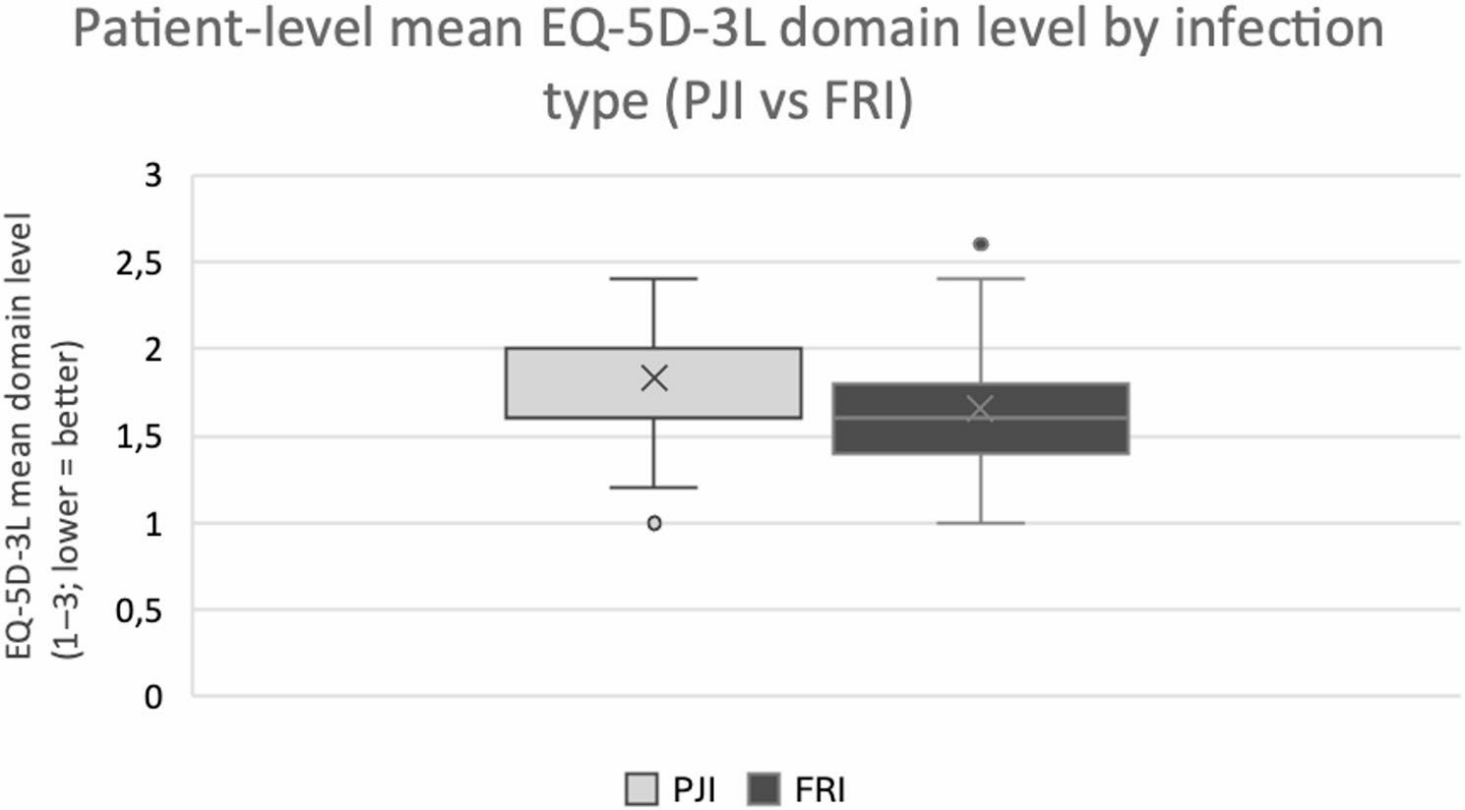
Patient-level mean EQ-5D-3 L domain level (average across the five dimensions; range 1–3, lower = better). Boxes show the interquartile range, horizontal line = median; “x” = group mean; whiskers = min-max; dot = outlier. Group sizes: PJI *n* = 22, FRI *n* = 26. Means ± SD: PJI 1.83 ± 0.37, FRI 1.65 ± 0.39; *p* = 0.061

The FRI group had a lower patient-level mean EQ-5D-3 L domain level (1.65 ± 0.39) than the PJI group (1.83 ± 0.37), indicating fewer reported problems. However, the difference was not statistically significant (*p* = 0.061). While means differed modestly, the distribution was skewed; medians were 1.6 (FRI) vs. 2.0 (PJI), Wilcoxon *p* = 0.061. Figure 3 shows the distribution across infection types (range 1-3; lower = better).

The American Society of Anesthesiologists (ASA) scores were significantly higher in the PJI group (mean: 2.71 ± 0.53; median 3) vs. FRI (mean 2.35 ± 0.55; median 2), Wilcoxon *p* = 0.018, *r* = 0.30. Most patients in the PJI group were classified as ASA 3, while most FRI patients were categorized as ASA 2; only a few patients in either group fell into ASA 1 or 4.

**Fig. 4:**
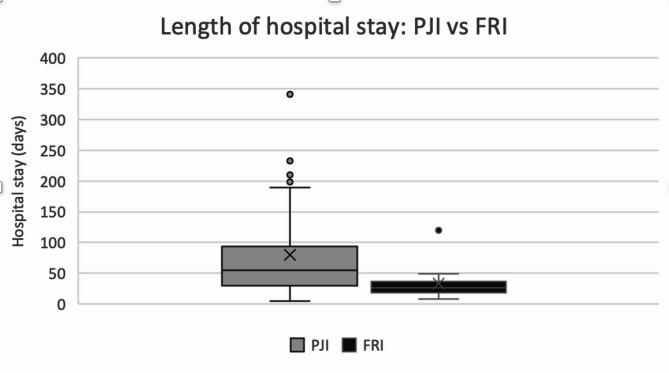
Length of hospital stay (days). Boxes show interquartile range; horizontal line = median; “x” = group mean; whiskers = min-max; dot = outlier. Group sizes: PJI *n* = 30, FRI = 31. Means ± SD: PJI = 82.9 ± 81.2 days, FRI = 33.1 ± 26.1 days; *p* < 0.001. Median (IQR): PJI 55 (31.5–87.0), FRI 27 (18.0–36.5)

Figure 4 shows that the average hospital length of stay was longer for patients with PJI (82.9 ± 81.2 days) than for those with FRI (33.1 ± 26.1 days), with a statistically significant difference (*p* < 0.001). The distribution was skewed, therefore median values are also reported: PJI 55 days (IQR: 31.5–87.0), FRI 27 days (IQR: 18.0–36.5). Group differences were large (Wilcoxon *p* < 0.001; *r* = 0.47), consistent with clinically meaningful skewed distributions.

On average, the time from index surgery to infection onset was longer in the PJI group (32.2 ± 59.0 months) than in the FRI group (12.7 ± 24.0 months). However, this difference was not statistically significant (*p* = 0.144).

The proportion of patients who underwent revision surgery was similar between the two groups (PJI: *n* = 17; FRI: *n* = 19; *p* = 1.000).

No significant group differences in pathogen distribution were observed. The most frequently isolated organisms in both the PJI and FRI groups were Staphylococcus epidermidis and Staphylococcus aureus (Table 1).

There were no significant group differences in terms of gender distribution (*p* = 0.300), reinfection rates (*p* = 0.281), amputation rates (*p* = 0.606), or smoking status ( *p* = 0.291). Recurrent infections after initial healing occurred in 18 patients in the PJI group and 14 patients in the FRI group, with no significant difference observed (*p* = 0.226) (Table 1).

There were no statistically significant differences between groups regarding family history of illness, presence of other comorbidities, educational background, or academic degree (Table 1). Effect sizes for the categorical variables were negligible to small (see Supplementary Table S 2).

Mortality until survey completion was 4/31 (12.9%) in the FRI group and 2/30 (6.6%) in the PJI group with no significant group difference (*p* = 0.672). All deaths occurred among non-responders; no survey respondents had died prior to questionnaire return.

In a multivariable linear regression model in which EQ-VAS was the dependent variable and the model was adjusted for age, sex ASA classification and time from the last index/revision surgery to the survey PJI (vs. FRI) was associated with an adjusted means difference of b = − 7.71 (95% confidence interval (CI): − 19.24 to + 3.82; *p* = 0.185). In an exploratory model that also included length of stay (LOS) this association was weaker (b = − 4.70; 95% CI – 17.02 to + 7.62; *p* = 0.446). This is consistent with the possibility that treatment intensity partly mediates the difference in HRQoL between PJI and FRI. Full regression outputs are provided in Supplementary Table S 1.

## Discussion

This study identified clinically and statistically significant differences between patients with periprosthetic joint infections (PJI) and fracture-related infections (FRI). PJI patients were older and had higher ASA classifications, longer hospital stays, and significantly lower EQ-VAS scores, which together suggest a greater overall burden of disease.

However, when multivariable regression analyses were adjusted for age, sex and ASA the observed difference in EQ-VAS between PJI and FRI decreased and became non statistically significant (b ≈ − 7.7, 95% CI – 19.2 to + 3.8). Including length of hospital stay in an exploratory model reduced the effect size further (b ≈ − 4.7, 95% CI – 17.0 to + 7.6). These findings suggest that some of the poorer HRQoL in PJI cases may be explained by baseline comorbidity and treatment intensity rather than by infection type alone.

The age difference may partly reflect epidemiological trends, given that total joint arthroplasty is more prevalent among older adults [21]. By contrast, fracture-related infections typically follow a bimodal distribution, peaking in younger males following high-energy trauma and in elderly females due to low-energy fragility fractures [22].

The advanced age and higher ASA scores in the PJI group may reflect reduced physiological reserves and greater comorbidity, factors that could contribute to delayed recovery and increased dependence. The extended hospital stay observed in the PJI group underscores the intensive nature of PJI treatment, which often involves multiple surgical procedures, prolonged antibiotic therapy, and an increased perioperative risk.

Hospitalisation itself can negatively affect quality of life through immobilisation, social isolation and psychological stress, which may partly explain the lower EQ-VAS scores in the PJI cohort.

As highlighted by Migliorini et al. (2021), complex cases may require advanced reconstructive strategies, such as the induced membrane technique or vascularised bone grafts [8]. These procedures are often necessary to restore mechanical stability and function following bone loss related to infection, and they contribute to the prolonged rehabilitation process. Moreover, Maffulli et al. (2016) stressed that the management of adult osteomyelitis relies on striking a balance between radical debridement and the preservation of viable tissue, principles that apply equally to both PJI and FRI management [9].

It is important to distinguish between the interval from index surgery to infection onset and the interval from index surgery to survey completion. While the former did not differ significantly between groups, the latter was markedly longer for patients with PJI, reflecting the chronic nature of the condition and the extended treatment trajectories that may negatively impact the long-term HRQoL.

While the patient-level mean EQ-5D-3 L domain level did not differ significantly between groups, EQ-VAS scores were lower than population norms in Germany [23].

Because we reported patient-level mean domain levels rather than EQ-5D-3 L utilities, direct comparison with EQ-5D-3 L utility normas is not possible and should be interpreted cautiously.

These results are consistent with previous studies indicating long-term impairments in physical function, self-care, and emotional well-being in patients recovering from implant-associated infections (10,14,15,23). Shichman et al. (2023) demonstrated that patients with PJI had significantly reduced mobility, self-care ability, and mental well-being compared to the control group [24].

These findings support the growing recognition of PJI as a chronic, systemically burdensome condition. Knebel et al. (2020) reported high levels of psychosocial distress, including anxiety and hopelessness, among PJI patients (14) and Krenn et al. (2025) emphasized the identity-disrupting effects of chronic PJI, which are often associated with social withdrawal and loss of autonomy [13, 14].

While we did not evaluate psychiatric comorbidities in our cohort, prior studies have reported higher rates of depression and anxiety in individuals with PJI, especially following revision surgery [2, 12]. Additionally, Browne et al. (2014) showed that pre-existing depression predicts poorer short-term outcomes following joint arthroplasty [11].

FRI is generally considered less severe; however, our findings and previous reports suggest that complex or recurrent cases can result in marked HRQoL impairments [5, 25]. He et al. (2023) emphasised the need for psychosocial support even in fracture-related infections, particularly when recovery is prolonged or complicated by reinfection [17].

Patients with delayed union or multiple surgical interventions may experience prolonged recovery and emotional distress. He et al. (2023) and Buijs et al. (2024) underscore the importance of structured psychosocial support for complicated FRI cases [17, 25].

Accordingly, international recommendations now increasingly advocate for multidisciplinary care approaches in FRI, similar to established strategies for PJI [26].

Notably, the HRQoL scores observed in our PJI cohort are comparable to those reported in patients with advanced oncological disease. While different instruments were used, the parallels underline the chronic, high-burden nature of musculoskeletal infections and the need for interdisciplinary care [20, 27, 28].

Even after successful infection control, many patients continue to experience lasting impairments. Zhang et al. (2022) and Cooper et al. (2024) confirmed that HRQoL remains compromised despite clinical remission. This underscores the need to differentiate between medical success and perceived recovery [15, 16]. Systematically including patient-reported outcomes in follow-up care is essential to address this gap.

Unlike in oncology, where psycho-oncological counselling and structured rehabilitation are standard components of care, such resources are often lacking in orthopaedic infection management [28, 29]. Closing this gap through standardised screening, psychosocial support, and personalized rehabilitation programmes could enhance long-term outcomes and patient satisfaction [11, 12].

Frameworks such as those developed by the International Consensus Meeting and the Pro-Implant Foundation already provide guidance on clinical stratification and treatment planning [30–32]. Expanding these models to integrate psychosocial assessment and long-term HRQoL monitoring would represent a crucial step toward truly patient-centred infection care.

### Limitations

This study has limitations inherent to its retrospective, single-center design, including potential selection and documentation biases. The small sample size (*n* = 61) limits statistical power and generalizability. The modest response rate (37.9%) may have introduced non-responder bias, although baseline characteristics were similar between responders and non-responders. Additionally, the absence of longitudinal follow-up precludes evaluation of changes in health-related quality of life (HRQoL) over time. While the EQ-5D is a validated instrument, it may not capture the full psychosocial burden experienced by this population.

Moreover, the use of patient-level mean EQ-5D-3 L domain levels instead of utilities limits comparability with some reference cohorts. Given baseline imbalances (e.g. age and ASA) residual confounding cannot be excluded despite exploratory regression analyses. Confidence intervals were wide for some comparisons and multiple testing increases the risk of type I error; results should therefore be interpreted with caution. Furthermore, anatomical sites differed between the groups (large joints in PJI vs. predominantly tibia/femur in FRI) which may limit direct comparability.

To better understand the patient experience, future research should incorporate prospective data, standardized mental health assessments and qualitative methods.

A matched-pair design should be considered for future studies to control for confounding factors such as age and ASA classification, enabling more direct comparisons between infection types.

Nevertheless, the study has notable strengths. These include microbiological confirmation of infection, consistent clinical documentation, and the use of a validated, preference-based HRQoL instrument. Comparing two clearly defined infection types within a unified institutional framework supports internal validity and strengthens the observed associations.

## Conclusion

Patients with periprosthetic joint infections have significantly lower health-related quality of life (HRQoL) than those with fracture-related infections. Although differences in EQ-5D-3 L domain levels did not reach statistical significance, EQ-VAS scores and overall HRQoL values were markedly reduced in PJI. Their outcomes are comparable in magnitude to those reported in advanced oncologic disease, highlighting the chronic and multifactorial nature of implant-associated infections. These findings emphasize the urgent need for integrated care pathways that extend beyond infection control. Systematic inclusion of patient-reported outcomes, structured psychosocial support and targeted rehabilitation programmes are essential for improving long-term outcomes. International consensus frameworks provide a solid basis for implementing patient-centered strategies in orthopedic infection care [7, 32].

## Supplementary Information

Below is the link to the electronic supplementary material.

Supplementary Material 1

## Data Availability

The data supporting the findings of this study are not publicly available due to institutional data protection policies and patient confidentiality. Data may be obtained from the corresponding author upon reasonable request and subject to ethical approval.

## References

[1] Rupp M, Walter N, Bärtl S, Heyd R, Hitzenbichler F, Alt V. Fracture-related infection- epidemiology, etiology, diagnosis, prevention, and treatment. Dtsch Arztebl Int. 2024;121:17–24. 37970721 10.3238/arztebl.m2023.0233 PMC10916768

[2] Walter N, Rupp M, Hinterberger T, Alt V. Prosthetic infections and the increasing importance of psychological comorbidities: an epidemiological analysis for Germany from 2009 through 2019. Orthopade. 2021;50:859–65. 33751197 10.1007/s00132-021-04088-7 PMC7942820

[3] Walter N, Rupp M, Lang S, Alt V. The epidemiology of fracture-related infections in Germany. Sci Rep. 2021;11:10443. 34001973 10.1038/s41598-021-90008-w PMC8128870

[4] Koutalos AA, Baltas C, Akrivos V, Arnaoutoglou C, Malizos KN. Mortality, functional outcomes and quality of life after hip fractures complicated by infection: a case control study. J Bone Jt Infect. 2021;6:347–54. 34611506 10.5194/jbji-6-347-2021 PMC8485839

[5] Iliaens J, Onsea J, Hoekstra H, Nijs S, Peetermans WE, Metsemakers WJ. Fracture-related infection in long bone fractures: a comprehensive analysis of the economic impact and influence on quality of life. Injury. 2021;52:3344–9. 34474918 10.1016/j.injury.2021.08.023

[6] Walter N, Rupp M, Hierl K, Pfeifer C, Kerschbaum M, Hinterberger T, et al. Long-term patient-related quality of life after fracture-related infections of the long bones. Bone Joint Res. 2021;10:321–7. 34008424 10.1302/2046-3758.105.BJR-2020-0532 PMC8160029

[7] Li C, Renz N, Trampuz A, Ojeda-Thies C. Twenty common errors in the diagnosis and treatment of periprosthetic joint infection. Int Orthop. 2020;44:3–14. 31641803 10.1007/s00264-019-04426-7 PMC6938795

[8] Migliorini F, La Padula G, Torsiello E, Spiezia F, Oliva F, Maffulli N. Strategies for large bone defect reconstruction after trauma, infections or tumour excision: a comprehensive review of the literature. Eur J Med Res. 2021;26. 10.1186/S40001-021-00593-9. 10.1186/s40001-021-00593-9 PMC8487570 34600573

[9] Maffulli N, Papalia R, Zampogna B, Torre G, Albo E, Denaro V. The management of osteomyelitis in the adult. Surgeon. 2016;14:345–60. 26805473 10.1016/j.surge.2015.12.005

[10] Wimalan B, Rupp M, Alt V, Walter N. The patients‘ perspective - a qualitative analysis of experiencing a fracture-related infection. Front Psychol. 2023;14:1126826. 37325738 10.3389/fpsyg.2023.1126826 PMC10267399

[11] Browne JA, Sandberg BF, D’Apuzzo MR, Novicoff WM. Depression is associated with early postoperative outcomes following total joint arthroplasty: a nationwide database study. J Arthroplasty. 2014;29:481–3. 24090662 10.1016/j.arth.2013.08.025

[12] Walter N, Rupp M, Hierl K, Koch M, Kerschbaum M, Worlicek M, et al. Long-term patient-related quality of life after knee periprosthetic joint infection. J Clin Med. 2021;10:907. 33668957 10.3390/jcm10050907 PMC7956307

[13] Knebel C, Menzemer J, Pohlig F, Herschbach P, Burgkart R, Obermeier A, et al. Peri-Prosthetic joint infection of the knee causes high levels of psychosocial distress: a prospective cohort study. Surg Infect (Larchmt). 2020;21:877–83. 32282286 10.1089/sur.2019.368

[14] Krenn VT, Bönigk MS, Trampuz A, Liebisch M, Perka C, Meller S. Coping with chronic periprosthetic joint infection after failed revision of total knee and hip arthroplasty: a qualitative study on patient’s experiences in treatment and healing. PLoS ONE. 2025;20:e0319509. 40073045 10.1371/journal.pone.0319509 PMC11902299

[15] Cooper D, Athan E, Yates P, Aboltins C, Davis JS, Manning L. How much does prosthetic joint infection and its successful treatment affect patient-reported quality of life? Clin Orthop Relat Res. 2025;483:183–93. 39466405 10.1097/CORR.0000000000003201 PMC11658756

[16] Zhang C, Liu Z, Lin Y, Cai Y, Zhang X, Huang Z, et al. Patient-reported outcome on quality of life and pain after revision arthroplasty for periprosthetic joint infection: a cross-sectional study. J Clin Med. 2022;11:7182. 36498756 10.3390/jcm11237182 PMC9741318

[17] He SY, Yu B, Jiang N. Current concepts of fracture-related infection. Int J Clin Pract. 2023;2023:4839701. 10.1155/2023/4839701 PMC10154639 37153693

[18] Rietbergen L, Kuiper JWP, Walgrave S, Hak L, Colen S. Quality of life after staged revision for infected total hip arthroplasty: a systematic review. HIP Int. 2016;26:311–8. 27443225 10.5301/hipint.5000416

[19] Moock J. Präferenzbasierte Lebensqualitätsmessung: der EQ-5D Fragebogen. Phys Medizin Rehabilitationsmedizin Kurortmedizin. 2008;18:245–9.

[20] Borchert K, Jacob C, Wetzel N, Jänicke M, Eggers E, Sauer A, et al. Application study of the EQ-5D-5L in oncology: linking self-reported quality of life of patients with advanced or metastatic colorectal cancer to clinical data from a German tumor registry. Health Econ Rev. 2020;10:1–14. 33313984 10.1186/s13561-020-00297-6 PMC7733616

[21] Kremers HM, Larson DR, Crowson CS, Kremers WK, Washington RE, Steiner CA, et al. Prevalence of total hip and knee replacement in the united States. J Bone Joint Surg Am. 2015;97:1386. 26333733 10.2106/JBJS.N.01141 PMC4551172

[22] Court-Brown CM, Caesar B. Epidemiology of adult fractures: a review. Injury. 2006;37:691–7. 16814787 10.1016/j.injury.2006.04.130

[23] Hinz A, Kohlmann T, Stöbel-Richter Y, Zenger M, Brähler E. The quality of life questionnaire EQ-5D-5L: psychometric properties and normative values for the general German population. Qual Life Res. 2014;23:443–7. 23921597 10.1007/s11136-013-0498-2

[24] Shichman I, Sobba W, Beaton G, Polisetty T, Nguyen HB, Dipane MV, et al. The effect of prosthetic joint infection on work status and quality of life: a multicenter, international study. J Arthroplasty. 2023;38:2685–e26901. 37353111 10.1016/j.arth.2023.06.015

[25] Buijs MAS, Haidari S, IJpma FFA, Hietbrink F, Govaert GAM. What can they expect? Decreased quality of life and increased postoperative complication rate in patients with a fracture-related infection. Injury. 2024;55:111425. 38402709 10.1016/j.injury.2024.111425

[26] Metsemakers WJ, Morgenstern M, Senneville E, Borens O, Govaert GAM, Onsea J, et al. General treatment principles for fracture-related infection: recommendations from an international expert group. Arch Orthop Trauma Surg. 2020;140:1013–27. 31659475 10.1007/s00402-019-03287-4 PMC7351827

[27] Huang W, Yang J, Liu Y, Liu C, Zhang X, Fu W, et al. Assessing health-related quality of life of patients with colorectal cancer using EQ-5D-5L: a cross-sectional study in Heilongjiang of China. BMJ Open. 2018;8:e022711. 30530472 10.1136/bmjopen-2018-022711 PMC6286482

[28] Lewandowska A, Rudzki G, Lewandowski T, Próchnicki M, Rudzki S, Laskowska B, et al. Quality of life of cancer patients treated with chemotherapy. Int J Environ Res Public Health. 2020;17:1–16. 10.3390/ijerph17196938 PMC7579212 32977386

[29] Kennedy IW, Haddad FS. Periprosthetic joint infection: navigating the literature. Bone Joint J. 2025;107–B:374–7. 40164189 10.1302/0301-620X.107B4.BJJ-2024-1080

[30] Izakovicova P, Borens O, Trampuz A. Periprosthetic joint infection: current concepts and outlook. EFORT Open Rev. 2019;4:482–94. 31423332 10.1302/2058-5241.4.180092 PMC6667982

[31] Parvizi J, Gehrke T, Chen AF. Proceedings of the International Consensus on Periprosthetic Joint Infection. Bone Joint J. 2013;95-B:1450–2. 10.1302/0301-620X.95B11.33135 24151261

[32] Cats-Baril W, Gehrke T, Huff K, Kendoff D, Maltenfort M, Parvizi J. International consensus on periprosthetic joint infection: description of the consensus process. Clin Orthop Relat Res. 2013;471:4065. 24155178 10.1007/s11999-013-3329-4 PMC3825924

