# Supplementary material for: Life after bone infection: a retrospective comparison of quality of life in patients with periprosthetic joint infection and fracture-related infections
Source: J Orthop Surg Res. 2025 Nov 19;20:1012. doi: 10.1186/s13018-025-06427-2 (PMC12628545; doi:10.1186/s13018-025-06427-2)

**Supplements:**

| **Model** | **Covariate** | **𝛽 (mean difference in EQ-VAS)** | **95% CI** | **p-value** |
| --- | --- | --- | --- | --- |
| **Model 1 (primary)** | PJI (vs. FRI) | -7.71 | -19.24 to +3.82 | 0.185 |
|  | Age (per year) | -0.20 | -0.67 to +0.26 | 0.385 |
|  | Female (vs. Male) | +5.03 | -6.74 to +16.81 | 0.394 |
|  | ASA (per class) | -9.27 | -20.58 to +2.03 | 0.106 |
| **Model 2 (exploratory incl. LOS)** | PJI (vs. FRI) | -4.70 | -17.02 to +7.62 | 0.446 |
|  | Age (per year) | -0.16 | -0.63 to +0.31 | 0.486 |
|  | Female (vs. Male) | +4.81 | -6.88 to +16.50 | 0.411 |
|  | ASA (per class) | -8.72 | -19.98 to +2.53 | 0.125 |
|  | LOS (per day) | -0.058 | -0.146 to +0.030 | 0.192 |

**Supplementary Table S 1:** Multivariable linear regression of EQ-VAS. Model 1 was pre-specified, adjusting for age, sex, ASA classification and time from last index/revision surgery to survey. Model 2 was exploratory additionally including length of stay (LOS) as a potential mediator. Results are based on complete-case analysis.

| **Variable** | **Test** | **p-value** | **Carmèr's V (adjusted)** | **Interpretation** |
| --- | --- | --- | --- | --- |
| Sex (females vs. male) | χ² | 0.364 | 0.08 | negligible |
| Side (left vs. right) | χ² | 0.301 | 0.11 | small |
| Reinfection (yes/no) | χ² | 0.281 | 0.11 | small |
| Recurrent infection after quiescence | χ² | 0.226 | 0.14 | small |
| Amputation (yes/no) | Fisher | 0.606 | 0.06 | negligible |

**Supplementary Table S 2:** Effect sizes for selected categorical comparison between FRI and PJI groups. Cramér’s V (bias-corrected) is reported as a measure of association strength (0.1 = small, 0.3 = medium, 0.5 = large). All values indicate negligible to small associations.

**Supplementary Material 1:** validated German version of EQ-5D-3L and EQ-VAS questionnaire (EuroQol Group 2009):


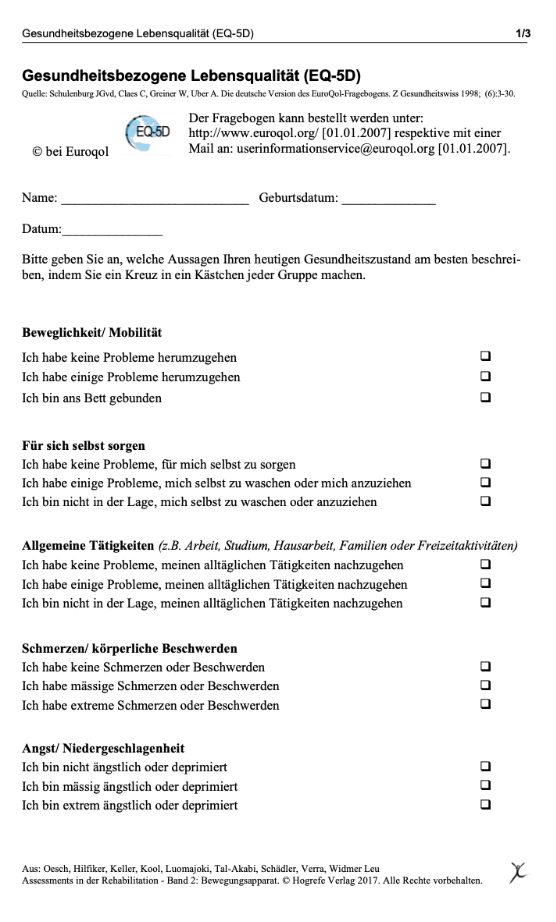

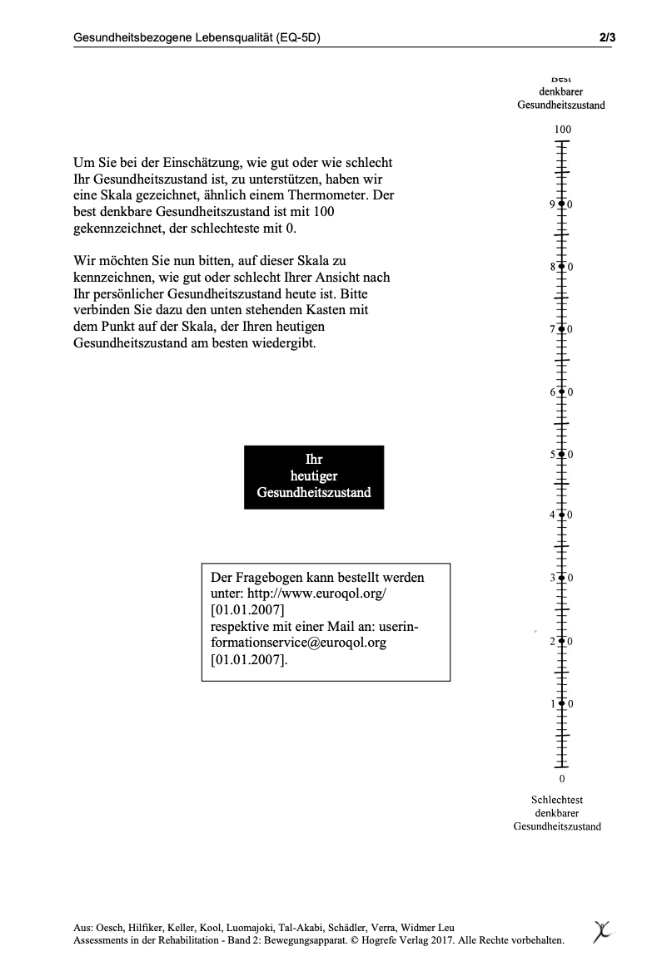

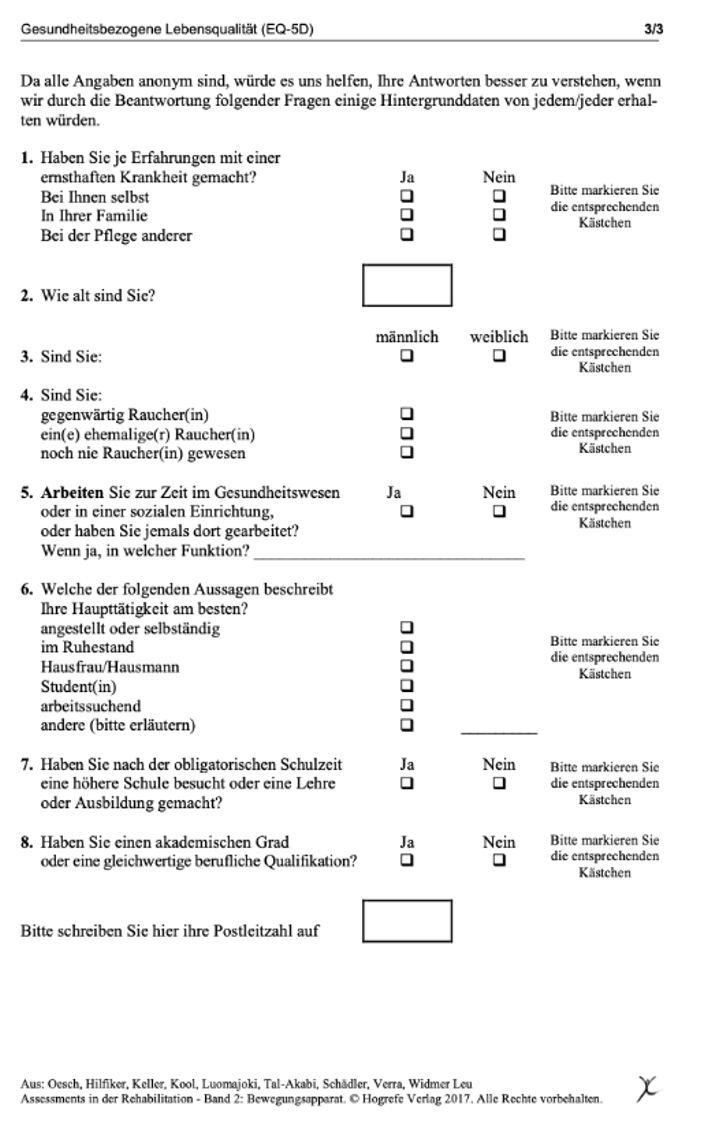

Supplement: Supplementary file 1 — Supplementary Material 1 [file 13018_2025_6427_MOESM1_ESM.docx]
